# CGUG: *in silico *proteome and genome parsing tool for the determination of "core" and unique genes in the analysis of genomes up to *ca*. 1.9 Mb

**DOI:** 10.1186/1756-0500-2-168

**Published:** 2009-08-25

**Authors:** Padmanabhan Mahadevan, John F King, Donald Seto

**Affiliations:** 1 Department of Bioinformatics and Computational Biology, George Mason University, 10900 University Boulevard, MSN 5B3, Manassas, VA, 20110, USA; 2 Current address: Department of Biological Sciences, Vanderbilt University, Nashville, TN 37235, USA; 3 Current address: Kingdomain Corporation, 10305 Nantucket Court, Fairfax, VA 22032, USA

## Abstract

**Background:**

Viruses and small-genome bacteria (~2 megabases and smaller) comprise a considerable population in the biosphere and are of interest to many researchers. These genomes are now sequenced at an unprecedented rate and require complementary computational tools to analyze. "CoreGenesUniqueGenes" (CGUG) is an *in silico *genome data mining tool that determines a "core" set of genes from two to five organisms with genomes in this size range. Core and unique genes may reflect similar niches and needs, and may be used in classifying organisms.

**Findings:**

CGUG is available at  as a web-based on-the-fly tool that performs iterative BLASTP analyses using a reference genome and up to four query genomes to provide a table of genes common to these genomes. The result is an *in silico *display of genomes and their proteomes, allowing for further analysis. CGUG can be used for "genome annotation by homology", as demonstrated with *Chlamydophila *and *Francisella *genomes.

**Conclusion:**

CGUG is used to reanalyze the ICTV-based classifications of bacteriophages, to reconfirm long-standing relationships and to explore new classifications. These genomes have been problematic in the past, due largely to horizontal gene transfers. CGUG is validated as a tool for reannotating small genome bacteria using more up-to-date annotations by similarity or homology. These serve as an entry point for wet-bench experiments to confirm the functions of these "hypothetical" and "unknown" proteins.

## Background

There is a tremendous increase in the number of genomes deposited in databases, with the data stream already a "data tsunami". The universal adoption of the "Next Generation" DNA sequencing technologies will also allow a parallel, expedited sequencing of smaller, but important and relevant, genomes such as from viruses and less than 2 Mb bacterial genomes.

Software tools for taking advantage of these data need to be developed as well as maintained and upgraded for additional and more useful functions. In particular, the readily available and "user-friendly" computational tools, preferably platform-independent, are especially needed as many wet-bench researchers are interested in the informational content, the "biology," of the genomes rather than the computational aspects of these genomes.

CGUG is a modification and extension of a web-based tool, CoreGenes [1], which was limited to genomes of viruses (ca. 350 kb), including chloroplasts and mitochondria. It now determines the "core" set of genes from a set of up to five bacteria with small genomes (~2 Mb). Its usefulness in the small genomes community has attracted researchers with diverse interests and needs. In response to some of these interests and needs, the tool has been upgraded with the input of wet-bench researchers.

While bacteria with larger genomes, ca. 4+ Mb, are of obvious importance, bacteria with genomes of smaller sizes are also of interest to the community; many of these are pathogens. Tools for data mining and analysis of the genomes and proteomes from these and other pathogens are important not only for understanding their basic biology, but also in the applications of these data for molecular surveillance and detection, including molecular diagnostics, as well as in drug design and discovery, including vaccine development.

For understanding the phylogeny of organisms, the determination of a set of common or "core" genes between a set of bacterial genomes provides insight into the particular and specific characteristics of those bacterial species and of their niches in the biosphere. Core genes are being used to reconstruct ancestral genomes [2], phylogenies [3] and organism classifications [4], and should provide insight into the common requirements of living in similar niches. The core set of genes has been used to explore the concept of the "pan-genome" of a bacterial species or a group of bacteria [5]. Essential genes comprising the minimal genome and the minimal life form, e.g., *Mycoplasma genitalium *[6] may be a subset of this core.

From a survey of the literature, there are relatively few tools for the determination of core genes from genomes. One example is CEGMA [7], which is used to annotate these in eukaryotic genomes. CEGMA is limited to the analysis of eukaryotic genomes. It is neither web-based nor functional across platforms, and must be downloaded and installed. Other tools have similar limitations or are confined to precomputed sets of genomes, or are no longer accessible/supported.

CGUG is a user-friendly "on-the-fly" web-based tool that determines, parses, analyzes and outputs a set of core genes from a set of two to five small bacterial genomes. As a validation of this tool, applications for analyzing *Chlamydophila *and *Francisella *genomes are presented, including reannotation, especially 'hypothetical proteins', illustrating the comparisons of newly-determined genomes with the analysis with older, less well-annotated genomes; that is, to align and to identify similar and also putatively similar proteins, previously noted as "unknown" and "hypothetical" entries. The current and future versions of this tool are available at .

In bacteriophage research, to complement the current classification criteria of the International Committee on the Taxonomy of Viruses (ICTV) [8] and to understand them better, a proteome tree analysis based on a BLASTP algorithm has been constructed earlier [9]. CGUG provides another independent in situ proteome analysis approach that incorporates suggestions by several ICTV members working on bacteriophages [4], noting that while these genomes contain horizontal transfers that have made understanding bacteriophage classification very difficult [4], a proteome-based approach can help to unravel and to understand their classifications [4].

## Implementation

### Algorithm

The algorithm is based on the GeneOrder algorithm to determine gene order and synteny [10]. GenBank accession numbers are inputted to select data files. These are extracted from GenBank and an iterative protein similarity analysis is performed for each protein from the query genome against the reference genome protein database using BLASTP from WU-BLAST.

### Limitations

Currently, CGUG is limited to the analysis of small bacterial genomes (up to 2 Mb). Furthermore, it is limited to the analysis of five genomes at a time. Both limitations are due to the computational power and allocated memory of our server, which frequently comes under heavy user load; we hope to migrate this tool to a more powerful server. But for now, this tool is limited by computational resources (i.e., hardware) that restrict the size and number of genomes that can be processed. However, during our test runs, 4 Mb genomes can be processed successfully. The caveat is that there is a significantly longer processing time (> 1 hr; there is a queuing e-mail return option). Despite these limitations, CGUG is a valuable tool for biologists and this has been illustrated by its use in the classification of bacteriophages [4].

#### Validation

*Chlamydophila *analysis of core genes; annotation application *Chlamydophila *(1 Mb "small" genomes) are interesting because some are responsible for causing diseases in humans and other mammals: *C. pneumoniae *is a respiratory pathogen that causes community-acquired pneumonia and bronchitis in humans [11]; *C. felis *causes conjunctivitis and upper respiratory tract disease in cats [12]; *C. abortus *causes abortions in ruminants such as sheep and goats [13]; and *C. caviae *causes conjunctivitis in guinea pigs [14]. Comparative genomics may provide insights into their biology as well as pathogenicity.

**Figure 1:**
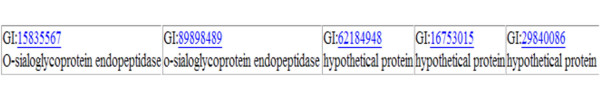
**A row of output from CGUG showing related proteins from five *Chlamydophila *genomes**. The annotated O-sialoglycoprotein endopeptidase in *C. pneumoniae *J138 and *C. felis *Fe/C-56, respectively, are noted to have identity to counterparts noted in three *Chlamydophila *genomes. These additional columns display the equivalent and presumably related proteins which have been annotated originally as "hypothetical" in *C. abortus*, *C. pneumoniae *AR39 and *C. caviae *GPIC. This provides a lead for additional bioinformatic analyses and wet-bench investigations.

**Figure 2:**
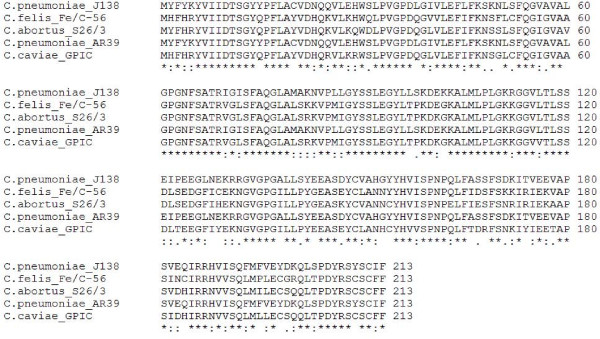
**Multiple sequence alignment of five proteins from *Chlamydophila *genomes**. The *C. pneumoniae *J138 and *C. felis *Fe/C-56 proteins displayed are annotated as O-sialoglycoprotein endopeptidase. CGUG analysis reveals counterpart proteins from *C. abortus *S26/3, *C. pneumoniae *AR39 and *C. caviae *GPIC that are annotated currently as "hypothetical proteins." As an example of additional bioinformatic analysis suggested by CGUG results, these counterparts are aligned to determine their identity to O-sialoglycoprotein endopeptidase. Conserved residues are indicated by asterisks. Colons indicate conserved substitutions, based on amino acid physico-chemical properties. Dots indicate semi-conserved substitutions.

As an example of the reannotation application, *Chlamydophila *genomes, Table 1, are analyzed for their core genes, yielding a set of 839 related proteins, with a stringency or threshold range setting of "75" (default). A visual inspection of this output reveals many hypothetical proteins across the genomes. By looking at a specific row of putatively related genes, a hypothetical protein in one genome can be identified or annotated by comparison with annotated proteins noted in the other genomes. Figure 1 displays proteins annotated as O-sialoglycoprotein endopeptidase in *C. pneumoniae *J138 and in *C. felis *Fe/C-56. The putatively related proteins in the same row are annotated as hypotheticals for *C. abortus *S26/3, *C. pneumoniae *AR39 and *C. caviae *GPIC. These must be analyzed further, as demonstrated in Figure 2 where CLUSTALW-based multiple sequence alignment (MSA) is presented. The extensive conserved residues and alignment suggest that the hypothetical proteins are likely O-sialoglycoprotein endopeptidases as well. Percent identities between the annotated proteins and the hypothetical proteins are relatively high, being 67% or greater, again, strongly suggests that these hypotheticals are O-sialoglycoprotein endopeptidases.

**Table 1:** Accession numbers and sizes of five analyzed *Chlamydophila *genomes

**Genome**	**Accession #**	**Size (Mb)**
*Chlamydophila pneumoniae *J138	NC_002491	1.23

*Chlamydophila felis *Fe/C-56	NC_007899	1.17

*Chlamydophila abortus *S26/3	NC_004552	1.14

*Chlamydophila pneumoniae *AR39	NC_002179	1.23

*Chlamydophila caviae *GPIC	NC_003361	1.17

*Francisella tularensis *SCHU S4	NC_006570	1.89

*Francisella tularensis holarctica*	NC_009749	1.89

*Francisella tularensis mediasiatica*	NC_010677	1.89

**Figure 3:**
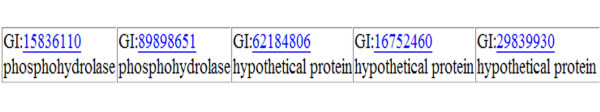
**Output of a row from CGUG showing phosphohydrolase-related proteins from five *Chlamydophila *genomes**. The first two columns display an annotated phosphohydrolase protein in *C. pneumoniae *J138 and *C. felis *Fe/C-56, respectively. The other three columns show related proteins from the CGUG result, annotated in the genome records as "hypothetical" for *C. abortus*, *C. pneumoniae *AR39 and *C. caviae *GPIC. This provides a lead for additional bioinformatic analyses and wet-bench investigations.

Another example is the annotation of a phosphohydrolase in *C. pneumoniae *J138 and in *C. felis *Fe/C-56; putatively related proteins are annotated as hypotheticals in other genomes, Figure 3. Percent identities between the annotated proteins and the hypothetical proteins are 63% or greater, suggesting a similar function. Further analyses must be performed to confirm this; that is, the ultimate assignments of function lie in wet-bench experiments as annotation by homology and similarity can *only suggest *function.

Genome annotation and methods for annotation have lagged behind the DNA sequencing technology, in part, due to the vast unknown of the biology and coding potential of organisms. Genomes that have been sequenced more recently take full advantage of newly accumulated knowledge, and therefore are annotated more completely and, presumably, with less error. For the non-computational biologist who is interested in the biology of related organisms, inspection and alignments of genomes annotated from different time periods may be problematic. CGUG allows older genomes to be matched with related and recently sequenced genomes.

### Application to the larger Francisella genomes

**Figure 4:**
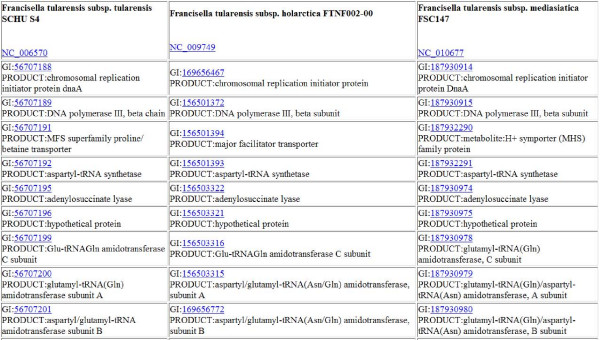
**Output of "core" set of proteins from three *Francisella *genomes**. Partial output of the "core" set of proteins from *Francisella tularensis *SCHU S4, *Francisella tularnensis holarctica *and *Francisella tularensis mediasiatica *are presented as an example of the core set of genes amongst these organisms. Each is linked to their GenBank record and may be retrieved for additional bioinformatic analyses.

**Figure 5:**
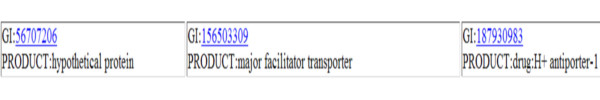
**Row of output from three *Francisella *genomes**. Counterpart proteins from *Francisella *genomes are displayed: the first column corresponds to *Francisella tularensis *SCHU S4; the second column corresponds to *Francisella tularnensis holarctica*; and the third column corresponds to *Francisella tularensis mediasiatica*. As noted in the text, the counterpart annotations provide a clue as to the function of the "hypothetical" protein, subject to additional bioinformatic analyses and wet-bench investigations.

*Francisella *genomes are larger, at approximately 1.89 Mb. Important pathogens are among them, e.g., *F. tularensis *causes tularaemia [15]. Three genomes, Table 1, are analyzed to determine their "core" set of proteins and to note the reannotation function of CGUG. These organisms share 1229 core proteins. Figure 4 shows the partial output of the core proteins table, revealing a hypothetical protein in *Francisella tularensis *SCHU S4 (published 2004). Annotated counterparts in the recently sequenced *Francisella tularensis holarctica *and *Francisella tularensis mediasiatica *FSC147 (2007) show this as a major facilitator transporter and drug:H+ antiporter-1, respectively (Figure 5). Percent identities between the hypothetical protein and these two annotations are 99.2% and 99.7%, strongly suggesting that the hypothetical protein is a transporter protein, again subject to validation by wet-bench confirmation.

### Bacteriophage classifications

Bacteriophages have been intensely studied in the laboratory, and their classifications have been debated and defined under current ICTV criteria, which include physical, clinical, biochemical and molecular data. Recently, several bacteriophage researchers have undertaken a re-evaluation of the bacteriophages given the availability of genome data and the in situ proteome data. This data analysis included parsing the numbers of shared similar and orthologous proteins, using both CoreGenes and CoreExtractor.vbs [4]. The majority of the accepted relationships and ICTV classifications have been re-confirmed for the *Podoviridae*, although several new insights appeared. One example, three established genera within the T7-related bacteriophages are reconfirmed, along with five putative novel genera. These proteome-inspired insights offer a refinement to the ICTV phage classification and provide a straightforward algorithm for the classification of new phage based on their genome and proteome [4]. The entire set of bacteriophages is being re-examined, beginning with the *Podoviridae*, above, and continuing with the *Myoviridae*, with plans for *Siphoviridae *and the rest.

As an example of CGUG analysis, bacteriophages from several genera of the *Microviridae *are analyzed in order to verify their current classification. These include Microvirus, Chlamydiamicrovirus, Bdellomicrovirus and Spiromicrovirus (Table 2). The first sequenced phage of each genus is used as the reference genome and is analyzed against the other members for shared similar proteins. A 40% cutoff for shared similar proteins is used for inclusion of a phage in a particular genus. This cutoff criterion has been used to verify the current classification of phages of the *Podoviridae *and to define novel genera as well, and also has been discussed in detail [4].

**Table 2:** Accession numbers and sizes of analyzed bacteriophage genomes

**Genome**	**Accession #**	**Size (bp)**
Enterobacteria phage α3	NC_001330	6087

Enterobacteria phage G4	NC_001420	5577

Enterobacteria phage ϕX174	NC_001422	5386

Enterobacteria phage S13	AF274751	5386

Enterobacteria phage ϕK	X60323	6089

*Chlamydia *phage 1	NC_001741	4877

*Chlamydia *phage 2	NC_002194	4563

*Chlamydia *pneumoniae phage CPAR39	NC_002180	4532

*Chlamydia *phage ϕCPG1	NC_001998	4529

*Bdellovibrio *phage ϕMH2K	NC_002643	4594

*Spiroplasma *phage 4	NC_003438	4421

Enterobacteria phage T7	NC_001604	39,937

Enterobacteria phage P22	NC_002371	41,724

Enterobacteria phage lambda	NC_001416	48,502

Using CGUG, *Chlamydia *phage 2 and *Chlamydia *phage ϕCPG1 share 50% similar proteins with *Chlamydia *phage 1. *Chlamydia pneumoniae *phage CPAR39 shares 42% similar proteins with *Chlamydia *phage 1. These values are above the shared protein cutoff of 40% and verify the current ICTV classification in the Chlamydiamicrovirus genus. Proteins unique to *Chlamydia *phage 1, with respect to the other phages, include several hypothetical proteins and proteins annotated as "structural proteins". Table 3 shows the percent identities and BLAST E-values between the shared proteins of *Chlamydia *phage 1 and *Chlamydia *phage ϕCPG1. Even though many of the percent identities are not very high, several of the E-values suggest a significance of alignments and relationships. Caveat: Need wet-bench experiments to confirm the functional properties.

**Table 3:** Percent identities and E-values between shared proteins of *Chlamydia *phage 1 and *Chlamydia *phage ϕCPG1

***Chlamydia *phage 1**	***Chlamydia *phage ϕCPG1**	**% identity**	**E-value**
VP1	hypothetical protein	49.0	e-160

VP2	capsid protein VP2-related protein	24.6	2e-25

VP3	capsid protein VP3	25.3	3e-13

hypothetical protein	nonstructural protein	60.0	2e-7†

hypothetical protein	hypothetical protein	18.9	7e-21

nonstructural protein	nonstructural protein	30.2	3e-8

The "†" indicates that the E-value was obtained with the low complexity filter in bl2seq turned off. This was done because the proteins are short (36 amino acids).

*Bdellovibrio *phage ϕMH2K, which belongs to the Bdellomicrovirus genus, shares significantly less than 40% similar proteins with the phages of the Microvirus genus. Specifically, it shares no similar proteins with ϕX174, G4 and ϕK. It only shares one protein with α3 and S13. *Bdellovibrio *phage ϕMH2K also shares less than 40% similar proteins with a phage of the Spiromicrovirus genus, *Spiroplasma *phage 4. These results justify the current separation of *Bdellovibrio *phage ϕMH2K from the Microvirus and Spiromicrovirus genera. In contrast, *Bdellovibrio *phage ϕMH2K shares approximately 45% similar proteins with the phages of the Chlamydiamicrovirus genera. There are discussions on merging these two genera; these *in silico *proteome results from CGUG lend more support to this position.

### Continuing development

Software development is an on-going process, both in terms of coding and hardware as well as research needs. CGUG is an example of this, being supported and updated in response to requests from researchers, e.g., reanalysis of all bacteriophages, and supported in regards to coding updates. A beta version (CGUG 3.1), at the same site, is an alternative and complementary upgrade that will continue to be improved. It provides a more robust user interface (UI) and aims to improve the user experience, including a time bar to monitor the run length. It provides for a better batch analysis, recommended especially for long running queries, such as for the 2 Mb genomes, and in preparation for the much larger bacterial genomes in the future, *ca*. > 4 Mb. Algorithm enhancements are needed and planned, as the current implementation does not handle these long running queries robustly. The feature list below summarizes anticipated current and continuing work:

• Improve user interface (UI)

◦ Show a dynamic status indicator of query progress

◦ Allow user to elect to receive results via email at any time

• Review implementation of algorithm for performance

• Add persistence (e.g., database) of queries and results by user

CoreGenes was originally designed for a nominal use case of a single query submission with the user waiting for the results page to be returned (synchronous mode); 3.1 now provides a better Batch Analysis mode option where the user provides their email address for subsequent delivery of results. The site is redesigned using Google Web Toolkit (GWT) technology, which is ideal for the requirements of a potentially long running response in a web-based application. GWT is based on Asynchronous JavaScript and XML (AJAX), which allows for a much more robust and interactive user experience in a browser-based application.

In this beta version (3.1) of CGUG, when the user submits a query, the web page indicates that the query has begun executing and will present the user with a query status indicator (e.g., a "progress bar"), with a message log. Once a query is submitted and has begun executing, the approximate number of iterations that will be required to complete the computation will be known. With minor modifications, the Java program that executes the query on the server will track the iterations completed and report back to the user progress via "call back" mechanisms that are easily implemented with GWT. Based on this, a rough "percent complete" indicator is displayed and will be updated continuously via a client side timer executing in JavaScript in the browser. Thus, the progress indicator will update automatically with no action required by the user, allowing for real-time updating.

## Conclusion

CGUG is an *in silico *genome and proteome data mining tool that is useful in the analysis of core genes from small-genome bacteria (~2 Mb), and in the putative assignments and suggestions of function for genes previously annotated as unknown or hypothetical, taking advantage of the new genomes and annotations as well as the growing databases for protein function assignment.

Another dimension of CGUG is realized in the reanalysis and verification of the current classifications of organisms, for example in the reanalysis and new insights of bacteriophages.

## Availability and requirements

Project name: CGUG

Project home page:  and general splash page,  (including version 3.1)

Operating system(s): Platform independent web-based

Programming language: Java, XML

Any restrictions to use by non-academics: License required for commercial usage

## Competing interests

The authors declare that they have no competing interests.

## Authors' contributions

PM implemented the software and performed the analyses. JFK provided additional ideas and coding. DS conceived the project. PM, JFK and DS wrote the manuscript. All authors read and approved the final manuscript.
